# *Regenerative Medicine Research*: striving to better serve the emerging field

**DOI:** 10.1051/rmr/160001-s

**Published:** 2016-08-12

**Authors:** Y. James Kang

**Affiliations:** 1 Regenerative Medicine Research Center, Sichuan University West China Hospital, Sichuan PR China; 2 Pharmacology and Toxicology, University of Louisville School of Medicine, Louisville USA

*Regenerative Medicine Research*, previously published by BioMed Central, is now transferred to its new publisher, EDP Sciences. As the Editor-in-Chief, I am excited to announce the continuation of this new open access, online journal. It remains to publish research relating to both the fundamental and practical aspects of regenerative medicine, with a particular emphasis on translational studies.

Regenerative medicine is an emerging field with the potential to empower medical research and practice. The process of tissue regeneration is essential to many organisms for a healthy life, from the highly studied axolotl, to us as human beings. In many ways regenerative medicine has become the leading edge of biomedical research and clinical practice, due to its key implications for the treatment of increasingly prevalent diseases, such as heart disease.

The field is in the infant stage of its development but it is growing at a fast pace. However, comprehensive information or research data on regenerative medicine cannot presently be efficiently gathered, but can only be scrutinized through a diversity of different journals in various fields. Regenerative medicine is, by current definition [1], the process of creating living, functional tissues to repair or replace tissue or organ function lost due to age, disease, damage, or congenital defects. Obviously, this requires a multidisciplinary approach, and one that looks beyond tissue engineering and stem cells. *Regenerative Medicine Research* just meets this need and continues to find its own niche in biomedical science.

As an open access journal, articles accepted for publication in *Regenerative Medicine Research* are made rapidly available online following acceptance. This ensures efficient dissemination of information and experimental data to scientific communities and the medical practice field. Importantly, a highly respected Editorial Board composed of internationally well-recognized experts in the field facilitates a fair, timely and rigorous peer review process.

The Editorial Board, Publisher, and myself assure our authors and readers that we are committed to making *Regenerative Medicine Research* a preeminent platform for exchanging research protocols and experimental data in this exciting, emerging field. We look forward to receiving your high quality contributions to the journal.

Editor-in-Chief
Y. James Kang
